# Minimization of the Wilson’s Central Terminal voltage potential via a genetic algorithm

**DOI:** 10.1186/s13104-018-4017-y

**Published:** 2018-12-20

**Authors:** Hossein Moeinzadeh, Paolo Bifulco, Mario Cesarelli, Alistair L. McEwan, Aiden O’Loughlin, Ibrahim M. Shugman, Jonathan C. Tapson, Aravinda Thiagalingam, Gaetano D. Gargiulo

**Affiliations:** 1 The MARCS Institute, Western Sydney University, Sydney, NSW Australia; 2 Department of Electrical Engineering and Information Technology (DIETI), “Federico II”, The University of Naples, Naples, Italy; 3 School of Electrical and Information Engineering, University of Sydney, Sydney, NSW Australia; 4 School of Medicine, Western Sydney University, Sydney, NSW Australia; 5 Cardiology Department, Campbelltown Hospital, Sydney, NSW Australia; 6 School of Medicine, The University of Sydney, Sydney, NSW Australia; 7 Cardiology Department, Westmead Hospital, Sydney, NSW Australia; 8 Westmead Institute for Medical Research, Sydney, NSW Australia; 9 School of Computing, Engineering and Mathematics, Western Sydney University, Sydney, NSW Australia

**Keywords:** Electrocardiography, Wilson Central Terminal, Genetic algorithm, Potential reference

## Abstract

**Objective:**

The Wilson Central Terminal (WCT) is an artificially constructed reference for surface electrocardiography, which is assumed to be near zero and steady during the cardiac cycle; namely it is the simple average of the three recorded limbs (right arm, left arm and left leg) composing the Einthoven triangle and considered to be electrically equidistant from the electrical center of the heart. This assumption has been challenged and disproved in 1954 with an experiment designed just to measure and minimize WCT. Minimization was attempted varying in real time the weight resistors connected to the limbs. Unfortunately, the experiment required a very cumbersome setup and showed that WCT amplitude could not be universally minimized, in other words, the weight resistors change for each person. Taking advantage of modern computation techniques as well as of a special ECG device that aside of the standard 12-lead Electrocardiogram (ECG) can measure WCT components, we propose a software minimization (genetic algorithm) method using data recorded from 72 volunteers.

**Result:**

We show that while the WCT presents average amplitude relative to lead II of 58.85% (standard deviation of 30.84%), our minimization method yields an amplitude as small as 7.45% of lead II (standard deviation of 9.04%).

**Electronic supplementary material:**

The online version of this article (10.1186/s13104-018-4017-y) contains supplementary material, which is available to authorized users.

## Introduction

The very first surface electrocardiogram was conceived and outlined by E. Einthoven in the 1900s, and entered clinical practice in the 1940s as 12-lead-Electrocardiogram (ECG). Since then, it is still the most popular non-invasive diagnostic tool for cardiac assessment [1, 2].

The 12-lead ECG is composed of twelve signals or ‘leads’ measured from the limbs and six positions on the chest called precordials. The precordials (1/2 of the signals) are measured as the potential difference between each exploring electrode located on the chest, and an assumed constructed ‘zero’ reference. This ‘zero’ reference was introduced by F. N. Wilson in 1931 and named after him as Wilson’s Central Terminal (WCT) [1]. By definition, it is the simple average of the three exploring electrodes connected to the right arm (RA), left arm (LA) and left leg (LL) and it is assumed to be steady and of negligible amplitude during the cardiac cycle.

However, the WCT voltage is neither steady nor of negligible amplitude [3]. Frank [4] was the first to undermine the idea of having a constant WCT during the cardiac cycle and discussed how this variation could affect the ECG measurement [2, 4–6]. Later, Burger clarified the true meaning of zero potential and defined the WCT as the average of the three limb leads which is symmetrical with respect to the limb leads [3]. To quantify the WCT voltage, Wilson proposed immersing the body in a large homogeneous conductor and theorized that 0.15 mV was its maximal value [7, 8].

Following this recommendation, Bayley and Kinard [9, 10] encased the body of volunteers inside a metal structure (called integrator electrode) that was immersed in water for the duration of the recording. With this experiment, they determined that the WCT is non-stationary during the cardiac cycle and its amplitude could be as large as 40% of Einthoven’s ECG signals [9, 10]. During this experiment, Bayley and Kinard also attempted a real-time minimization of the WCT amplitude to bring it below a non-influent value [10]. To achieve minimization they made use of three rheostats instead of fixed resistors and adjusted the weights of the three WCT components continually reporting the achieved new amplitude. To Achieve the WCT recording for the minimization, the volunteer was encased by a metal structure that was submerged in the water for the duration of the recording [10].

Although the notion that a large WCT voltage may be affecting the clinical recordings, aside from the few notable research studies [11, 12], all recording methods currently employed use the raw WCT as a reference for precordials. Eventually, after an initial wave of interest, the WCT has received scant research attention during the past decades [2]. Recently Gargiulo et al. [2, 12] proposed a way to record unipolar ECG without using the WCT and suggested a new device and a method to measure and store the WCT components. Taking advantage of the availability of these unique recording we present a software minimization for the WCT.

Our method similarly to the originally attempted minimization performs a weighted average of the WCT components. To achieve this goal we use a genetic algorithm (GA) [13]. The GA is a heuristic search method for finding the optimal answer for problems with high computational complexity. This approach is used for those problems, like ours, that either lacks a deterministic solution or a deterministic polynomial time complexity solution. This algorithm is called “genetic” because is based on the concept of the biological evolution of individuals within a population where “chromosomes” mutates to achieve the survival of the fittest. A “chromosome” represents a possible solution to the problem that can mutate from one population of chromosomes to the next population (generation) by using a “selection” procedure. The chromosomes that are selected to be in the next generation also could be changed or become parents of new chromosomes in the process of “mutation”, and “crossover” [13]. In this paper, we show results of our minimization method applied to data recorded from 72 patients (25 female, age average 66.35 year-old ± 11.46 year-old), at Campbelltown Hospital, New South Wales, Australia.

## Main text

To our knowledge, this is the first attempt to the WCT minimization employing new data since Bayley and Kinard’s effort in 1954 [10]. The proposed approach to estimate M-WCT (whereas M stands for minimized) is engineered to fulfill:

1. Possibly be zero or near zero;
2. In any case (worst case scenario), M-WCT amplitude should be less than 0.1 mV, so that it can be considered clinically irrelevant and smaller than Wilson estimation of max amplitude [14].

In order to fit the M-WCT into the genetic algorithm paradigm, we need to formalize our problem regarding population, mutation, crossover and fitness function. Recalling that the WCT is the average of the limbs’ electrodes, we can define M-WCT as the weighted mean of the WCT’s components (weights can be different from 1/3, positive, not null and add up to one). More formally:

1.1 $$ M\text{-}WCT = \alpha \emptyset_{L} + \beta \emptyset_{R} + \gamma \emptyset_{F} $$

1.2 $$ 0 < \alpha ,\beta ,\gamma < 1 $$

1.3 $$ \alpha + \beta + \gamma = 1 $$

whereas $\alpha ,\beta , \;{\text{and}}\;\gamma$ are the minimization parameters; $\emptyset_{L} , \emptyset_{R}$ and $\emptyset_{F}$ are the raw potential of the limb electrodes placed on LA, RA, and LL respectively; thus $\alpha ,\beta , \;{\text{and}}\;\gamma$ are related to LA, RA and LL respectively, and will replace the averaging 5 kΩ resistors in our minimization.

To summarize, our method minimizes (1.1) according to the three main criteria enounced above constrained by (1.2) and (1.3).

### Fitness function

**Fig. 1:**
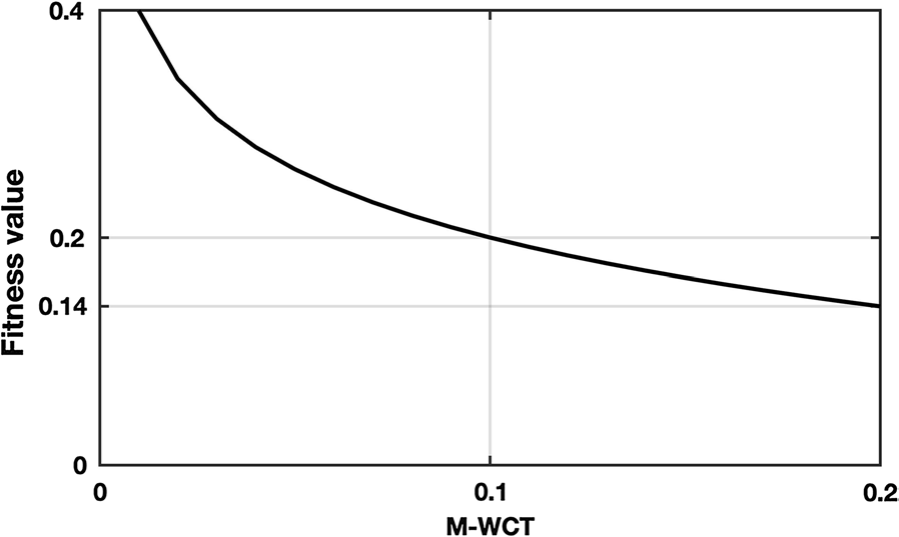
Nonlinear fitness function is used to encourage individuals to have smaller M-WCT

As mentioned, the role of the fitness function is to ensure “the survival of the fittest” hence converging towards the solution. Equation (1.4) is selected as the fitness function. This is because its shape and its nonlinearity provide an increase in the probability of having M-WCT with smaller values; in other words, it encourages the algorithm to converge more rapidly to amplitudes smaller than 0.1 mV. The plot of the fitness function is depicted in Fig. 1.

1.4 $$ {\text{Fitness}} = \log_{0.00001} \left| {M\text{-}WCT} \right| $$

### Population

Represents all possible answers (chromosomes), a new population is generated during each iteration where each individual is a tern of weighted factors $\left( {\alpha ,\beta ,\gamma } \right)$. 80 individuals are chosen as population size. In the first population, only one chromosome is initialized by the WCT chromosome ($\alpha = \beta = \gamma$ = 1/3) and the rest are generated randomly constrained only by the conditions (1.2) and (1.3). Also, (1) the elite members of each population are moved to the next generation directly; (2) the WCT chromosomes are preserved in each population.

### Crossover

The role of the crossover operation is to build the next population based on selected chromosomes of the current population. We use single point and averaging crossover methods randomly to populate the next generation. For details see [15, 16].

### Mutation

To avoid trapping into local optima the permutation algorithm is used to change the position of three parameters in a selected chromosome (mutation probability is equal to 0.1).

This method is applied to every voltages sample of the recording and computes three parameters to have the M-WCT trace during the cardiac cycle. For this study, we included data from 72 patients; each selected data excerpt has a normalized length of 10 s. As mentioned earlier, three weighting factors $\alpha ,\beta , \;{\text{and}}\;\gamma$ are calculated using the GA to minimize Wilson Central Terminal. In Additional file 1: Table S1, we report the average values of the three weights and the average number of iterations that the GA needs to converge for all patients.

**Fig. 2:**
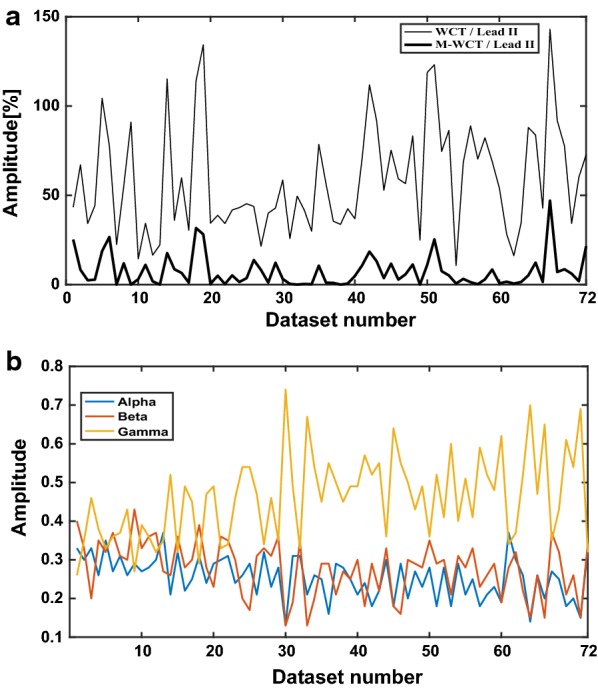
Direct comparison of the WCT and M-WCT (**a**); average of weighing parameters (**b**). **a** Direct comparison of M-WCT (bold trace) and WCT relative to lead II amplitude. **b** Trend of the three (average across the full 10 s) weighting parameters

Similarly to previous studies [9, 10], the WCT and M-WCT amplitudes are measured averaging five consecutive beats for each dataset and reported as a percentage of lead II. We used the orientation of the QRS complex to report the polarity of the WCT. “N” denotes signals with an unclear polarity in which the positive deflection amplitude closely matched the negative deflection at the QRS. In Fig. 2, we report the amplitude of the M-WCT and the WCT for each patient as well as the average across the full 10 s of the three weighting parameters. As it can be seen in Fig. 2b, the variation of each parameter is too high among all the patients, and three optimal parameters cannot be found for all the patients. Summary of our findings are summarized in Additional file 1: Table S1.

**Fig. 3:**
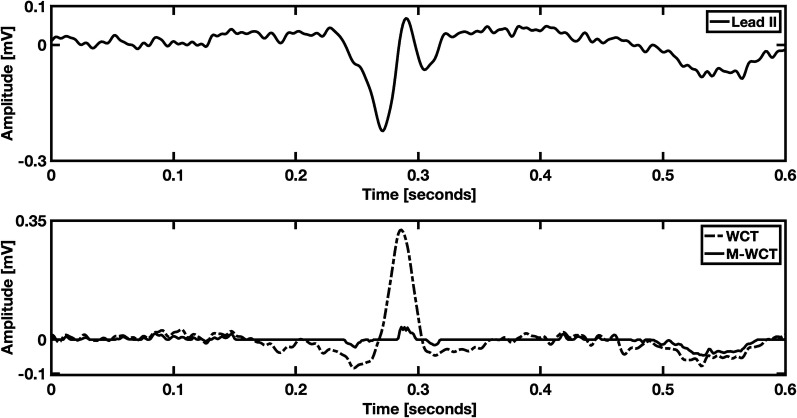
Example of positive deflection WCT. M-WCT is 11.04% of lead II amplitude, while WCT is 118.82% of lead II amplitude (average); the recording is from a 73-year-old male patient admitted at the hospital

Similarly, to previous studies, we found a variety of the WCT shapes and polarities (slightly in favor, see Additional file 1: Table S1, for positive polarity) hence it is possible to say that the WCT is highly individual, and can have standard ECG characteristics, such as a P-wave and a T-wave. Individuality was also found in the M-WCT, as we show in Fig. 3. However, due to the negligible general amplitude of M-WCT (see Fig. 2), we conclude that the clinical impact of the M-WCT is negligible with respect to the WCT.

Figure 3 shows an example of WCT in which a broader QRS feature with amplitude even higher than Lead II. However, as one can infer comparing M-WCT and WCT (bottom panel), the relative amplitude of M-WCT has highly decreased compared to the WCT relative amplitude.

An example of the WCT signal with a marked T-wave is visible in Additional file 2: Figure S1. As it can be observed, a marked T-wave deflection on the WCT trace (bottom panel) is synchronized with the T-wave on lead II (top panel). The WCT trace in Additional file 2: Figure S1 is also an excellent example of a highly variable WCT and an almost steady M-WCT. In one single cardiac cycle, the deflection’s polarity of the WCT changes at least three times, and its amplitude reaches 45.27% of lead II (average), while the M-WCT amplitude is 3.59% of lead II. Additional file 3: Figure S2 is an example of high WCT amplitude with negative deflection. As seen, the WCT has an amplitude of 59.21% of lead II, while M-WCT amplitude is only 2.79% of lead II.

Recall that there was an attempt to measure and minimize the WCT in 1954 with a peculiar experiment which required a very cumbersome setup [9, 10, 17]. Minimization was attempted by varying the weight resistors connected to the limbs in real time, and it showed that the WCT amplitude could not be universally minimized (see Additional file 4: Figure S3). They found out the value of the WCT is zero for half of their subjects when the three resistors were chosen as $(r = l = 2.6f)$. Unfortunately, this resistors selection, for the other tested subjects) decreased the WCT amplitude to less than 50% when compared to an unweighted terminal selection $\left( {r = l = f} \right)$ [17]. Our results (see Fig. 2b) shows that to minimize WCT amplitude, different weights should be used similarly to Bayley et al. experiment [17]. Additionally, we found that $\gamma$ (the weighting factor of $\emptyset_{F}$) have usually larger amplitudes in comparison with $\alpha$ and $\beta$. However, as the WCT component signals ($\emptyset_{L} , \emptyset_{R}$ and $\emptyset_{F}$) are highly individual, the computed weighted factors are also different for each patient. In our dataset, the ratio of $\gamma$ to $\alpha$ is in the range of [0.78, 5.69], and the ratio of $\gamma$ to $\beta$ is in the range of [0.62, 5.69] (see Fig. 2).

## Limitations

Although with this work we overcome the need to submerge the patients in water and use manual rheostats actually achieving a real minimization, probably the largest limitation is represented by the need to collect an excerpt of data that need to be used for the GA. For this work, we used a data excerpt of 10 s that at a sample rate of 800 Hz means a buffer for 8000 samples. Although this may seem like a small number, considering that to converge our GA takes 200 ± 41 iterations requiring few minutes on an average computer before that the new precordials can be computed adding considerable delay to the ECG diagnosis. To improve our method, we are currently working on faster minimization techniques that could make the use of M-WCT viable in clinical practice.

## Additional files

**Additional file 1: Table S1.** Measurements summary.

**Additional file 2: Figure S1.** Example of negative deflection WCT. WCT is 59.21% of lead II amplitude, while M-WCT is 2.79% of lead II amplitude (average); the recording is from a 59-year-old male patient admitted with chest pain.

**Additional file 3: Figure S2.** Example of negative deflection WCT. WCT is 59.21% of lead II amplitude, while M-WCT is 2.79% of lead II amplitude (average); the recording is from a 59-year-old male patient admitted with chest pain.

**Additional file 4: Figure S3.** Comparison of three resistors for right hand, left hand, and foot electrodes for 33 patients, experiment done by Bayley and Schmidt.

## Abbreviations

WCT: Wilson Central Terminal
ECG: electrocardiogram
GA: genetic algorithm
RA: right arm
LA: left arm
LL: left leg
M-WCT: Minimized Wilson Central Terminal
