# Supplementary material for: Minimization of the Wilson’s Central Terminal voltage potential via a genetic algorithm
Source: BMC Res Notes. 2018 Dec 20;11:915. doi: 10.1186/s13104-018-4017-y (PMC6302462; doi:10.1186/s13104-018-4017-y)
Supplement: Supplementary file 1 — Additional file 1: Table S1. Measurements summary. [file 13104_2018_4017_MOESM1_ESM.docx]

| N=72 | Age | Gender | WCT polarity | Alpha | Beta | Gamma | WCT | M-WCT | GA Iteration |
| --- | --- | --- | --- | --- | --- | --- | --- | --- | --- |
| Average | 66.35 y.o |  |  | 0.26 | 0.28 | 0.46 | 58.85% | 7.45% | 199.39 |
| Standard Deviation | 11.46 y.o |  |  | 0.05 | 0.07 | 0.11 | 30.84% | 9.04% | 41.18 |
| Other |  | F=35% | +: 55.5% N: 4.2% |  |  |  |  |  |  |
