# Supplementary figures and images for: Minimization of the Wilson’s Central Terminal voltage potential via a genetic algorithm
Source: BMC Res Notes. 2018 Dec 20;11:915. doi: 10.1186/s13104-018-4017-y (PMC6302462; doi:10.1186/s13104-018-4017-y)

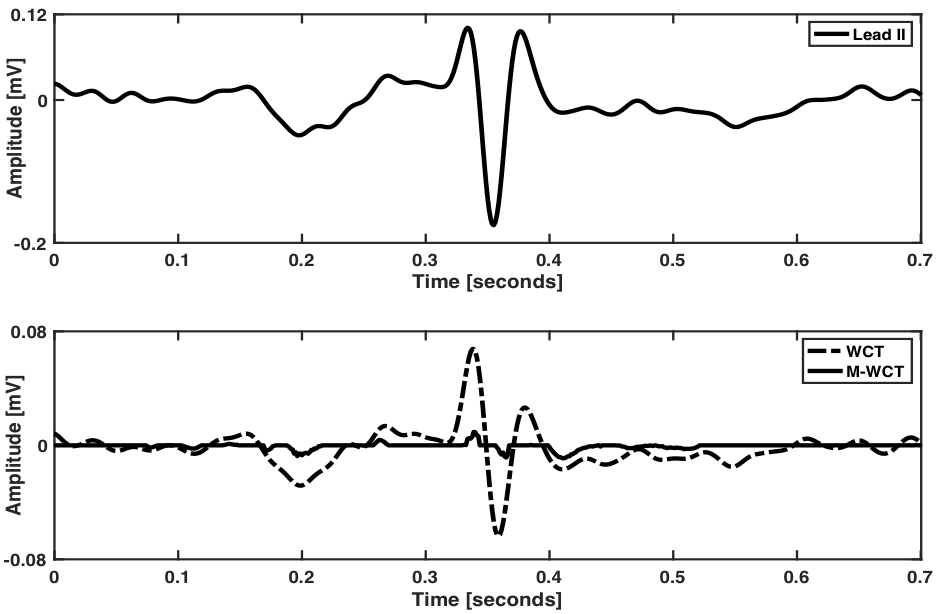

Supplement: Supplementary file 2 — Additional file 2: Figure S1. Example of negative deflection WCT. WCT is 59.21% of lead II amplitude, while M-WCT is 2.79% of lead II amplitude (average); the recording is from a 59-year-old male patient admitted with chest pain. [file 13104_2018_4017_MOESM2_ESM.png]

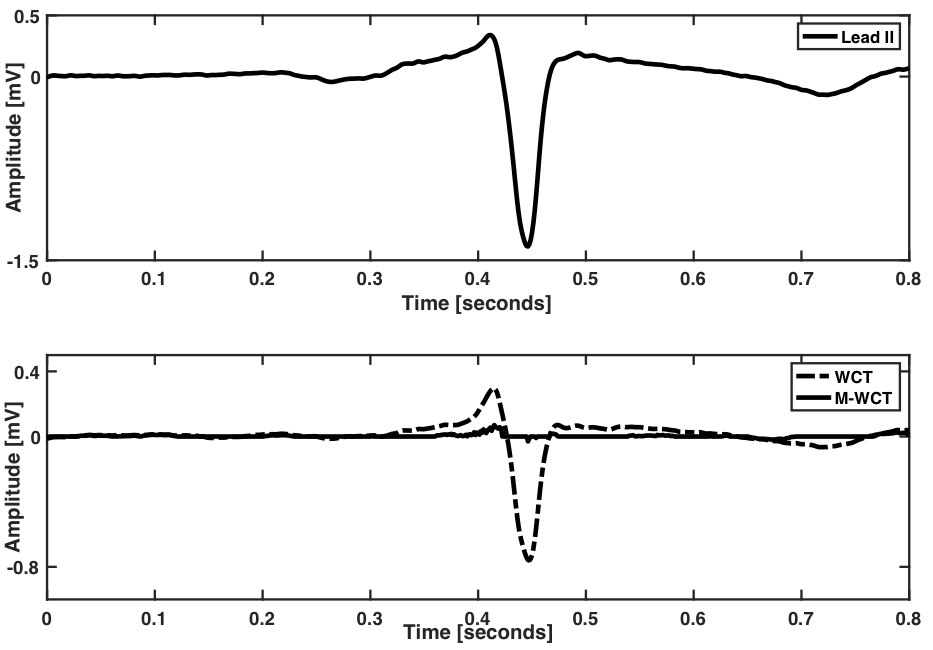

Supplement: Supplementary file 3 — Additional file 3: Figure S2. Example of negative deflection WCT. WCT is 59.21% of lead II amplitude, while M-WCT is 2.79% of lead II amplitude (average); the recording is from a 59-year-old male patient admitted with chest pain. [file 13104_2018_4017_MOESM3_ESM.png]

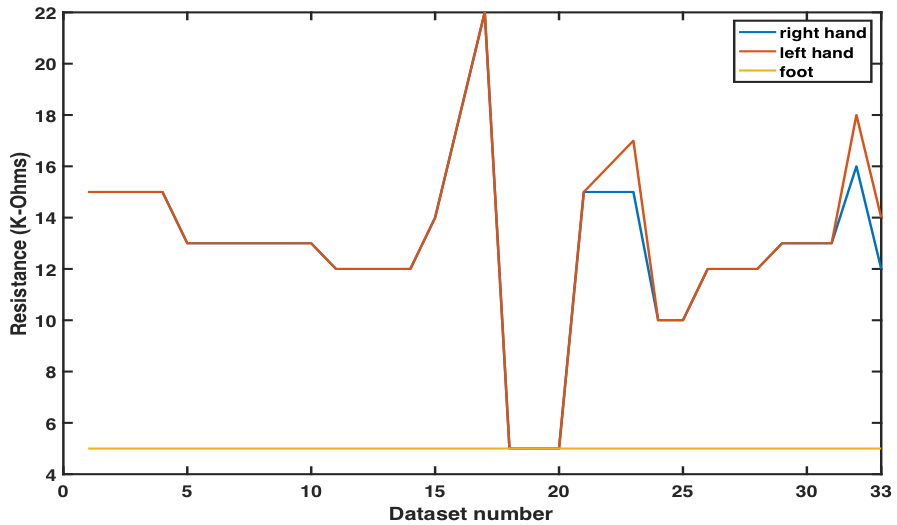

Supplement: Supplementary file 4 — Additional file 4: Figure S3. Comparison of three resistors for right hand, left hand, and foot electrodes for 33 patients, experiment done by Bayley and Schmidt. [file 13104_2018_4017_MOESM4_ESM.png]
